# MicroRNA-31 and MicroRNA-155 Are Overexpressed in Ulcerative Colitis and Regulate IL-13 Signaling by Targeting Interleukin 13 Receptor α-1

**DOI:** 10.3390/genes9020085

**Published:** 2018-02-13

**Authors:** Markus Gwiggner, Rocio T. Martinez-Nunez, Simon R. Whiteoak, Victor P. Bondanese, Andy Claridge, Jane E. Collins, J. R. Fraser Cummings, Tilman Sanchez-Elsner

**Affiliations:** 1 Clinical and Experimental Sciences, Sir Henry Wellcome Laboratories, University of Southampton School of Medicine, Southampton SO17 1BJ, UK; markus.gwiggner@uhs.nhs.uk (M.G.); r.t.martinez-nunez@soton.ac.uk (R.T.M.-N.); srw299@doctors.org.uk (S.R.W.); victorpaky@yahoo.com (V.P.B.); andrew.claridge1@nhs.net (A.C.); j.e.collins@soton.ac.uk (J.E.C.); 2 University Hospital Southampton NHS FT, Tremona Road, Southampton SO16 6YD, UK; fraser.cummings@uhs.nhs.uk; 3 School of Immunology and Microbial Sciences. MRC-Asthma UK Centre in Allergic Mechanisms of Asthma, Guy’s Campus King’s College, London SE1 9RT, UK

**Keywords:** ulcerative colitis, microRNAs, interleukin-13, inflammation, epithelium, gut

## Abstract

Interleukin-13 (IL-13) is an important Type 2 T helper (Th2) cytokine, controlling biological functions in epithelium and has been linked to asthma, atopic dermatitis and ulcerative colitis (UC). Interleukin-13 signals through IL-13 receptor α-1 (*IL13RA1* (gene) and IL13Rα1 (protein)), a receptor that can be regulated by microRNAs (miRs). MicroRNAs are small non-coding single-stranded RNAs with a role in several pathologies. However, their relevance in the pathophysiology of UC, a chronic inflammatory condition of the colonic mucosa, is poorly characterised. Here, we determined the expression of IL13Rα1 in UC, its potential regulation by miRs and the subsequent effect on IL-13 signalling. Inflamed mucosa of UC patients showed decreased mRNA and protein expression of *IL13RA1* when compared to healthy controls. We show that miR-31 and miR-155 are upregulated in inflamed UC mucosa and that both directly target the 3′ untranslated region of *IL13RA1* mRNA. Transfection of miR-31 and miR-155 mimics reduced the expression of *IL13RA1* mRNA and protein, and blocked IL-13-dependent phosphorylation of signal transducer and activator of transcription 6 (STAT6) in HT-29 cells, a gut epithelium cell line. Interleukin-13 activation of suppressor of cytokine signaling 1 (*SOCS1*) and eotaxin-3 (*CCL26)* expression was also diminished. MicroRNA-31/microRNA-155 mimics also downregulated *IL13RA1* in ex vivo human inflamed UC biopsies. We propose that miR-31 and miR-155 have an important role in limiting IL-13 signalling in UC disease.

## 1. Introduction

Interleukin-13 is a Type 2 T helper (Th2) cytokine that is believed to be required for normal immune function, such as defence against gastrointestinal nematodes and intracellular infections [1]. It has been suggested to have a role in mucosal inflammation and fibrosis in chronic diseases, including asthma, atopic dermatitis, eosinophilic oesophagitis and ulcerative colitis (UC) [1,2]. 

Ulcerative colitis and Crohn’s disease represent the two main types of inflammatory bowel disease [3]. Ulcerative colitis is a relapsing, idiopathic, chronic inflammatory condition of the colonic mucosa in genetically predisposed individuals [4,5] who are believed to mount an inappropriate immune response to their gut microflora or other environmental agents [6]. 

Interleukin-13 producing cells, including natural killer T cells (NKT) and macrophages, can be found in the healthy and non-inflamed human lamina propria [7,8]. Low levels of IL-13 may be required for homeostasis but IL-13 expression may be induced by infection or injury [1]. Studies in UC have reported increased release of IL-13 from activated lamina propria mononuclear cells [9] and non-classical NKT cells [10], with the latter having cytotoxic potential in cultured epithelial cells. Moreover, cultured colonic epithelial barrier models treated with IL-13 showed increased epithelial permeability, with apoptosis and increased expression of claudin 2, recapitulating changes seen in active UC [9,11,12]. 

The main mechanism of IL-13 signalling is via a protein dimer of IL-13 receptor α-1 (*IL13RA1* (gene) and IL13Rα1 (protein)) and IL-4 receptor α (IL4RA), eliciting phosphorylation of the signal transducer and activator of transcription 6 (STAT6) via Janus kinases (JAK) [13]. Interleukin-13 receptor α-1 is expressed in colonic epithelium of both healthy mucosa and mucosa affected by UC [11], however its expression in UC has not been quantified. Our group has shown that *IL13RA1* mRNA is directly targeted by microRNA (miR)-155 leading to downregulation of IL-13-driven gene expression in macrophages [14]. 

MicroRNAs are single-stranded short (~22 nt) non-coding RNAs that inhibit the translation and/or promote the degradation of their target mRNAs through binding to their 3′ untranslated region (3′UTR) [15], altering expression levels and biological function [16]. Human gut mucosal biopsies from inflammatory bowel disease have shown differential miR profiles compared to normal tissue [17,18,19,20,21,22,23,24,25,26]. Thus, studying disease-related changes in miR expression may give insight into underlying pathophysiological mechanisms in colonic epithelial inflammation. 

Given the differential expression of miRs in inflammatory bowel disease [17,18,19,20,21,22,23,24,25,26] potentially targeting *IL13RA1* [27], and the importance of IL-13 in the gut mucosa [1,10,11], we set out to investigate the expression levels of *IL13RA1* in UC and the possible role of miRs in its regulation. Our work demonstrates that IL13Rα1 is downregulated in inflamed mucosa from patients with UC facilitated by miRs. MicroRNA-31 and miR-155 were able to reduce IL-13 signalling in gut epithelial cells through downregulation of expression of IL13Rα1. These data give new insight into the regulation of the IL-13 pathway by miRs in UC.

## 2. Materials and Methods

### 2.1. Characteristics of Patients

Informed consent was obtained from patients with active UC undergoing lower gastro intestinal (GI) endoscopy as part of their routine clinical care for up to eight additional biopsies to be taken (Southampton and South West Hampshire Research Ethics Committee (A), reference number: 10/H0502/69). Patient paired samples with distal disease were identified from our tissue bank who had a partial endoscopic Mayo score (based on endoscopic evaluation) of 2–3 in the inflamed active segment and a paired corresponding biopsy (Mayo score 0–1) from the non-affected sigmoid area of the same patient, were used to analyse mRNA and miR expression matching them to the normal (unpaired) controls. All control samples were collected from normal colonic tissue in the sigmoid colon of patients who attended for colonic polyp surveillance (Table 1, Table 2, Table 3 and Table 4). 

### 2.2. Cell Culture

HT-29 and HeLa cells were cultured in Dulbecco’s modified eagle medium (DMEM) 10% fetal calf serum (FCS) (Thermo Fisher Scientific, Waltham, MA, USA). Colonic biopsies were placed in Aqix RS-1 (Aqix, London, UK) for ex vivo experiments or snap frozen in liquid nitrogen. 

### 2.3. Epithelial Cell Isolation

Isolation of epithelial cells from colonic samples was done in epithelial isolation buffer (EBI, pH 7.3) containing: 27 mM Trisodium citrate, 5 mM Na_2_HPO_4_, 96 mM NaCl, 8 mM KH_2_PO_4_, 1.5 mM KCl, 0.5 mM dithiothreitol (DTT), 55 mM d-Sorbitol and 44 mM Sucrose. RNAse inhibitor (400 U/mL) and phosphatase inhibitors were freshly added to the buffer. Five biopsies per patient were taken from the sigmoid colon and snap frozen in liquid nitrogen. Samples were then transferred into 1 mL of EBI at 4 °C in a cold room on dry ice. Samples were inverted gently by hand until detachment of the epithelial cells was observed and then again gently shaken. To detach cells from the crypts, the samples were gently vortexed 3 times for 10 s to free intact or partially broken crypts from the underlying matrix. Samples were then spun at 4 °C at 3000 rpm for 5 min. Pelleted cells (epithelium) were harvested in TRI reagent (Thermo Fisher Scientific).

### 2.4. Reverse Transcription and Real-Time PCR

RNA was extracted using TRI reagent (Thermo Fisher Scientific) according to manufacturer’s instructions. Colonic biopsies were disrupted in TRI reagent using a MagNA Lyser (Roche, Basel, Switzerland) and SiLibeads (Sigmund Lindner, Oldham, UK) prior RNA extraction. Reverse transcription was performed using high capacity complementary DNA (cDNA) reverse transcription kit following manufacturer´s instructions (Thermo Fisher Scientific). Random hexamer primers were used for cDNA generation and specific miR primers for miR analysis. Real-time PCR (qPCR) was performed in a ABI 7900HT fast real-time PCR system (Thermo Fisher Scientific) using TaqMan^®^ universal PCR master mix, No AmpErase^®^ Uracil N-Glycosylase (UNG). mRNA expression was detected using TaqMan^®^ gene expression assays and miR expression using TaqMan^®^ miR assays according to manufacturer’s instructions. Glyceraldehyde-3-phosphate dehydrogenase (GAPDH) and small nucleolar RNA C/D Box 44 (RNU44) primers were used as normaliser housekeeping genes for mRNA and miR analyses, respectively. All reverse transcription and real-time PCR (RT-qPCR) reagents were purchased from Thermo Fisher Scientific.

### 2.5. Cloning and Dual Luciferase Experiments

The genomic region encompassing miR-31 was amplified by PCR from genomic DNA (gDNA) using AmpliTaq gold DNA polymerase (Thermo Fisher Scientific), subcloned into pCR2.1 TOPO-TA cloning kit (Thermo Fisher Scientific) and then into pCDNA3.1(-) (Thermo Fisher Scientific). Primers employed were: miR-31_FOR: (*Xho*I) CTC GAG CAC TGA AGA GTC ATA GTA TTC TCC; and miR-31_REV: (*Hin*dIII) AAG CTT AAA TCC ACA TCC AAG GAA GGG CG. The reporter for the 3′UTR of *IL13RA1* containing the potential binding site for miR-31 was previously generated [14]. Mutation of the binding site of miR-31 was done using QuickChange site directed mutagenesis (Stratagene, San Diego, CA, USA) following the manufacturer’s protocol. Primers employed were: IL13RA1_3′UTR_MUT1_FOR: CTG CTA CTC AAG TCG GTA CCA CTG TGT CTT TGG TTT GTG CTA GGC CCC; and IL13RA1_3′UTR_MUT1_REV: GGG GCC TAG CAC AAA CCA AAG ACA CAG TGG TAC CGA CTT GAG TAG CAG. Transfections for the dual Luciferase experiments were done in HeLa cells using Superfect (Qiagen, Hilden, Germany) and assayed employing the dual Luciferase reporter assay (Promega, Madison, WI, USA) following manufacturer’s instructions.

### 2.6. Western Blotting

Cells were lysed in 1% NP-40 and complete protease inhibitor cocktail (Roche). Protein quantification was done using bicinchoninic acid (BCA) Assay (Pierce, Thermo Fisher Scientific) following manufacturer´s instructions. Electrophoresis was done under reducing conditions using the NuPAGE^®^Novex system (Thermo Fisher Scientific) and transfer of the samples was performed using XCell SureLock^®^ MiniCell and Xcell II™ blot module kit (Thermo Fisher Scientific) into polyvinylidene fluoride (PVDF) membranes. Blocking of the PVDF membranes was done in 2% ECL prime blocking agent (GE Healthcare, Buckinghamshire, UK). Antibodies used were: anti-IL13Rα1 (sc27861, Santa Cruz Biotechnology, Dallas, TX, USA), anti-β actin antibody loading control (ab8227, Abcam, Cambridge, UK) and anti-phosphoSTAT6 (#9361 Cell Signalling Technology, Danvers, MA, USA). Protein visualization was done using the ECL Select^TM^ Western blot detection reagent (GE Healthcare) in a VersaDoc (Bio-Rad Laboratories, Hercules, CA, USA). Densitometry was performed using Quantity One software (Bio-Rad laboratories).

### 2.7. Pre-miR Transfections

HT-29 cells were transfected with 100 nM Pre-miR™ miR precursors (Negative control#1, miR-31, miR-155 or a combination of 50 nM miR-31 + 50 nM miR-155, Thermo Fisher Scientific) using Interferin (Polyplus, New York, NY, USA) following manufacturer’s instructions. For IL-13 stimulation experiments, cells were stimulated 24 h post-transfection with 100 ng/mL IL-13 (R&D Systems, Minneapolis, MN, USA) and harvested 24 h later.

### 2.8. Biopsy Explants Culture and Pre-miR Transfection

Four sigmoid biopsies from each active UC patient with a Mayo score greater than 2 were preserved in Aqix RS-1 (Aqix). Samples were transferred onto a 96 well plate (U-bottom) in 200 μL of Aqix solution and transfected with 100 nM Pre-miR™ miRNA precursors (Negative control #1, miR-31, miR-155 or a combination of miR-31/155 mix (50 nM each)) using Interferin (Polyplus) following manufacturer’s instructions. Explants were then incubated under regular conditions (37 °C and 5% CO_2_) for 24 h. RNA was then isolated and RT-qPCR was carried out. 

### 2.9. Statistical Analysis

Paired *t*-test (parametric or non-parametric) were used for the analysis of paired unaffected and inflamed colonic samples (mRNA and miR). Comparison to normal controls was done employing unpaired tests. Unpaired Mann–Whitney test (one sided) was performed for Western blot statistics. All statistical analysis was performed using GraphPad Prism version 6.00 for Windows (GraphPad Software, La Jolla, CA, USA).

## 3. Results

### 3.1. IL13Rα1 Expression Is Downregulated in Actively Inflamed Ulcerative Colitis

**Figure 1:**
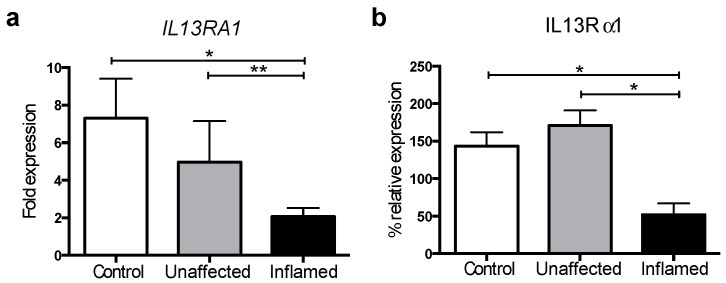
Interleukin-13 receptor α-1 (*IL13RA1* (gene) and IL13Rα1 (protein)) expression is dysregulated in ulcerative colitis (UC): (**a**) Bar graph depicting the mRNA expression of *IL13RA1* in colonic biopsies from non-UC patients (Control), unaffected tissue (Unaffected) and their matched inflamed colonic tissue samples taken from the same patient (Inflamed) relative to Control (*n* = 11 on each group); and (**b**) Bar graph depicting the relative expression of IL13Rα1 protein in colonic biopsies from non-UC patients (Control), unaffected tissue (Unaffected) and their matched inflamed colonic tissue samples taken from the same patient (Inflamed) relative to Control (*n* = 6 for each group). Data were first assessed for normality employing a D’Agostino and Pearson omnibus test when possible. Statistics were then done employing two-sided *t*-tests for non-parametric data (paired or unpaired): two-sided Mann–Whitney U-tests for non-paired datasets and Wilcoxon matched-pairs signed rank test for paired non-parametric data. Represented are mean + standard error of the mean. *: *p*-value ≤ 0.05; **: *p*-value ≤ 0.01.

Given that IL-13 signalling depends on the binding to the IL13Rα1 subunit [13], we first investigated the expression of *IL13RA1* in UC. We collected colonic biopsies from UC patients (paired inflamed and uninflamed tissue, see Table 1) as well as healthy controls (patients undergoing endoscopy for a polyp or cancer surveillance). We then extracted RNA and performed RT-qPCR. Interleukin-13 receptor α-1 mRNA expression was found downregulated in biopsies from inflamed tissue compared to uninflamed tissue as well as compared to healthy donor controls (Figure 1a).

We validated these findings at the protein level using Western blot analysis in an independent set of samples (Table 2), which showed that IL13Rα1 protein levels were also downregulated when comparing inflamed colonic tissue to unaffected or healthy controls (Figure 1b and Figure S1). These results reveal that IL13Rα1 is downregulated in the inflamed mucosa of UC patients.

### 3.2. MicroRNAs -31 and -155 Are Upregulated in Inflamed Tissue from Active UC Patients

**Figure 2:**
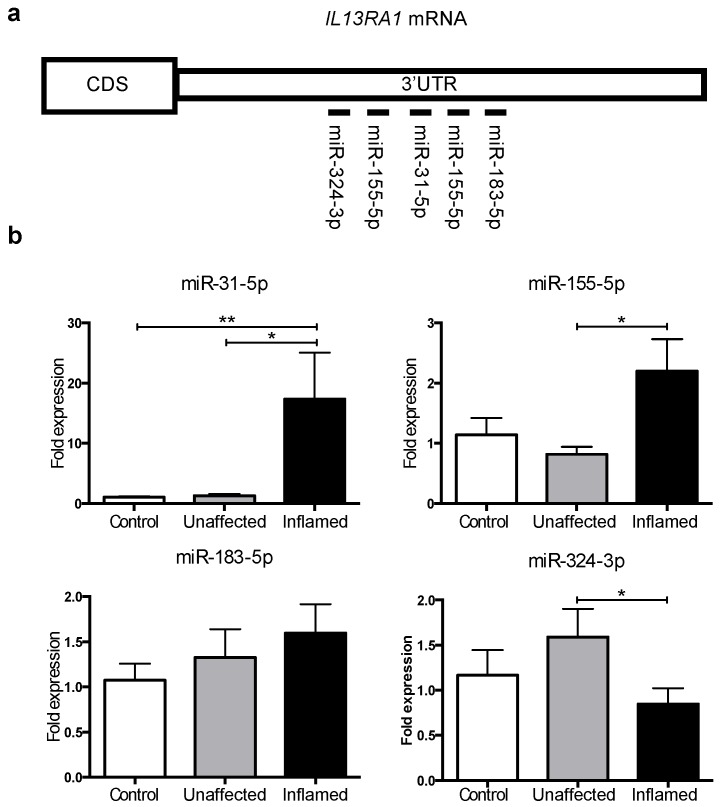
MicroRNAs predicted to target *IL13RA1* mRNA expression levels in UC colonic biopsies: (**a**) Schematic of the 3′UTR of *IL13RA1* mRNA representing the approximate locations for predicted (validated for miR-155) miR binding sites (Table S1); and (**b**) Bar graphs depicting the expression levels of miR-31-5p, miR-155-5p, miR-183-5p and miR-324-3p in colonic biopsies from non-UC patients (Control), unaffected tissue (Unaffected) and their matched inflamed colonic tissue samples taken from the same patient (Inflamed) (*n* = 7 on each group) relative to Control. Represented are mean + standard error of the mean. Data were normalised to expression of small nucleolar RNA C/D Box 44 (RNU44) prior to performing one-sided *t*-tests (paired for Unaffected vs. Inflamed or unpaired when comparing against Control). *: *p*-value ≤ 0.05; **: *p*-value ≤ 0.01.

MicroRNAs can inhibit gene expression by affecting mRNA stability and/or translation into protein [15]. Given the downregulation of *IL13RA1* mRNA and protein expression in inflamed colonic biopsies from patients with UC, we hypothesised that miRs may regulate this process. We employed TargetScan [27] to define putative miR binding sites on the 3′UTR of *IL13RA1*. Amongst miRs identified to potentially target *IL13RA1*, we selected a subset previously reported to be dysregulated in inflammatory bowel disease (Figure 2a and Table S1): miR-155-5p [28,29], miR-31-5p [28,30], miR-183-5p [28] and miR-324-3p [30]. None of these previous reports, however, have compared healthy, inactive UC and active UC patients and some of these miRs were only detected in Crohn's disease, rather than in UC. These miRs were assessed by RT-qPCR in endoscopic biopsies of inflamed UC and corresponding uninflamed tissue from the same patient and their expression levels determined relative to healthy donors. Figure 2b shows that only miR-31-5p and miR-155-5p (hereinafter miR-31 and miR-155, respectively) expression was significantly increased in inflamed UC tissue compared to unaffected samples. 

Our data indicate that miR-31 and miR-155 upregulation may contribute to the downregulation of *IL13RA1* in inflamed mucosa in UC. Importantly, this is the first time that miR-31 expression has been shown to be overexpressed in active UC patients compared to inactive UC or healthy donors.

### 3.3. miR-31 Directly Targets IL13RA1 mRNA

**Figure 3:**
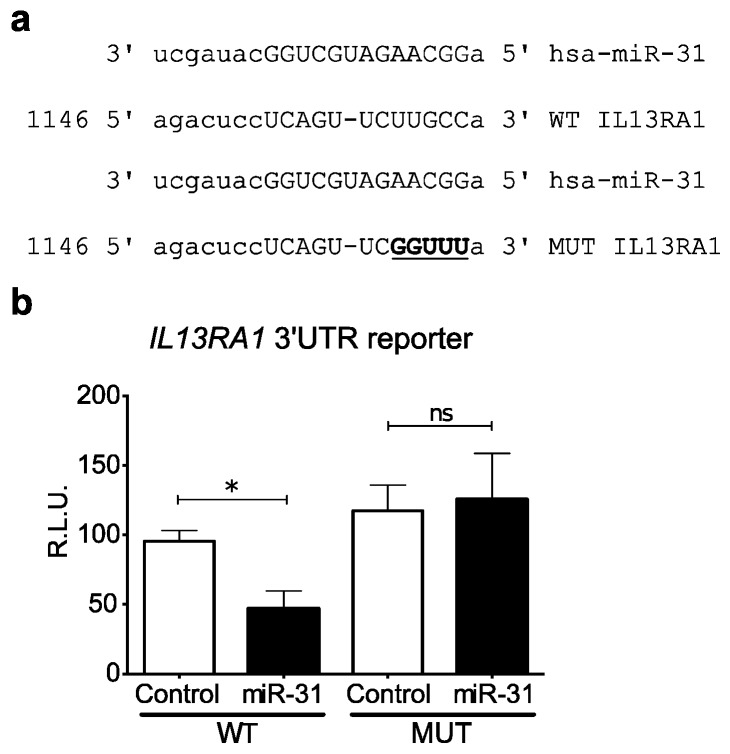
MicroRNA-31 directly binds and targets the 3′UTR of *IL13RA1* mRNA: (**a**) Schematic depicting the predicted binding site for miR-31 in the 3′UTR of *IL13RA1* mRNA (WT) and the generated mutant version (MUT); and (**b**) Bar graph showing the effects of over expressing miR-31 on the enzymatic activity of the Luciferase reporter generated for the predicted binding site and its mutant version. Co-transfection of miR-31 with the WT and MUT reporters in HeLa cells determined that miR-31 was able to significantly decrease the activity in the WT reporter only. Statistics were done employing a paired two-tailed *t*-test. Represented are mean + standard error of the mean R.L.U.: Relative luminescence units. *: *p*-value ≤ 0.05.

We have previously shown that miR-155 directly targets *IL13RA1* mRNA [14]. In silico prediction [27] indicated that miR-31 directly binds to the 3′UTR of *IL13RA1* (Figure 2a and Figure 3a). We therefore tested the direct binding of miR-31 to *IL13RA1* 3′UTR mRNA by employing a dual luciferase reporter assay. We generated a reporter construct (Figure 3a) fusing a renilla luciferase reporter gene to the 3′UTR of *IL13RA1* containing the sequence for the predicted binding site of miR-31 (wild type (WT) in Figure 3a). By performing site directed mutagenesis, we generated a mutant version for the predicted binding site of miR-31, with the aim of abrogating binding (mutant (MUT) in Figure 3a). We transfected HeLa cells, since this is a cellular system with generally less endogenous interference (lower levels of miRs and targets). Co-transfection of the renilla reporter *IL13RA1* 3′UTR constructs together with a miR-31 expression vector (or control empty vector) showed that over expression of miR-31 significantly reduced luciferase activity of the WT reporter, while it did not affect the activity of the mutated reporter (Figure 3b). These results show, for the first time, that miR-31 directly targets *IL13RA1* mRNA, binding to the sequence comprising nucleotides 1158–1165 on its 3′UTR.

### 3.4. IL13RA1 Expression Is Reduced in Primary Inflamed Ulcerative Colitis Gut Epithelium with Increased miR-31 and miR-155 Levels

**Figure 4:**
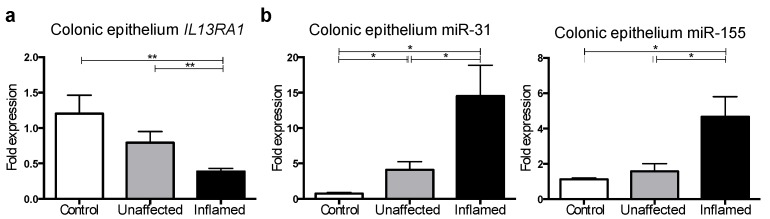
Interleukin-13 receptor α-1, miR-31 and miR-155 expression in primary gut epithelial cells: (**a**) Expression levels of *IL13RA1* mRNA in colonic epithelial cells extracted from patients gut biopsies from non-UC patients (Control), unaffected tissue (Unaffected) and their matched inflamed colonic tissue samples taken from the same patient (Inflamed) relative to Control (*n* = 5 on each group); and (**b**) miR-31 and miR-155 expression levels in gut epithelial cells isolated from biopsies taken from non-UC patients (Control, *n* = 3), unaffected tissue (Unaffected, *n* = 5) and their matched inflamed colonic tissue samples taken from the same patient (Inflamed, *n* = 5) relative to Control. Represented are mean + standard error of the mean. *: *p*-value ≤ 0.05; **: *p*-value ≤ 0.01.

The colonic epithelium serves as a barrier and regulates immune homeostasis [31] in the gut mucosa. Interleukin-13 increases apoptosis of colonic epithelial cells and contributes to the disruption of the epithelial colonic barrier in UC [9,11]. Given the direct-targeting of *IL13RA1* by both miR-31 (Figure 3) and miR-155 [14], we assessed the expression of *IL13RA1* and these miRs in isolated epithelial cells from colonic biopsies comparing healthy controls with unaffected and inflamed mucosa from UC patients (Table 3). Interleukin-13 receptor α-1 mRNA expression was found to be decreased in epithelium from inflamed tissue compared with unaffected tissue as well as healthy controls (Figure 4a). As expected, miR-31 and miR-155 were found to be upregulated (Figure 4b) in these cells, consistent with their potential to regulate *IL13RA1* expression. 

### 3.5. MicroRNA-31 and miR-155 Reduce IL-13 Signalling by Downregulating IL13Rα1 Expression in Gut Epithelial Cells

Given the upregulation of miR-31 and miR-155 in biopsies from colonic mucosa and isolated epithelial cells in active UC, while *IL13RA1* expression is decreased in active UC (Figure 1, Figure 2 and Figure 4) and the direct-targeting of *IL13RA1* mRNA by miR-31 and miR-155 (Figure 3 [14]), we hypothesised that these miRs may directly affect *IL13RA1* levels and IL-13 signalling in colonic epithelium. 

**Figure 5:**
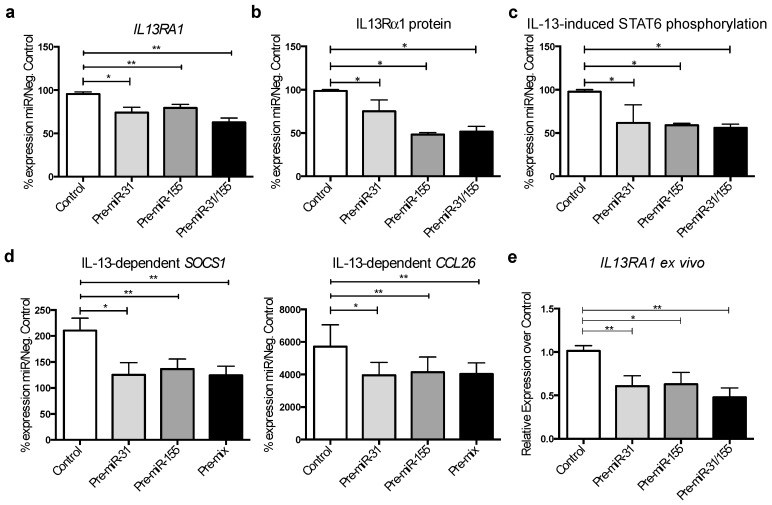
MiR-31 and miR-155 regulate the expression of IL13Rα1 in gut epithelium: (**a**) Bar graph representing the % of relative expression of *IL13RA1* mRNA (left, *n* = 9); and (**b**) Protein (right, *n* = 3) after transfecting HT-29 cells with miR control, miR mimics for miR-31, miR-155 or a combination of both miR-31 and miR-155; (**c**) Bar graph depicting the densitometry results for IL-13-dependent phospho-signal transducer and activator of transcription 6 (STAT6) in HT-29 cells transfected with miR control, miR mimics for miR-31, miR-155 or a combination of both miR-31 and miR-155 (*n* = 3); (**d**) Bar graph depicting the IL-13-dependent expression of suppressor of cytokine signaling 1 (*SOCS1)* and eotaxin-3 (*CCL26)*mRNA in HT-29 cells transfected with miR control, miR mimics for miR-31, miR-155 or a combination of both miR-31 and miR-155 (*n* = 9); and (**e**) Bar graph depicting the mRNA expression of *IL13RA1* in inflamed UC biopsies transfected with miR control, miR mimics for miR-31, miR-155 or a combination of both miR-31 and miR-155 (*n* = 7), relative to negative control. Represented are mean + standard error of the mean. Statistics were done employing *t*-tests *: *p*-value ≤ 0.05; **: *p*-value ≤ 0.01.

Human colonic epithelial cells (HT-29) were used as a tested tool in studying the role of IL-13 and miRs [32]. Cells were transfected with miR mimics (Pre-miR-31 and Pre-miR-155) individually (100 nM) or in combination (50 nM each) and compared to 100nM negative scrambled control mimics (Control). Twenty-four hours after transfection, *IL13RA1* mRNA and protein expression was assessed using RT-qPCR and Western blotting, respectively. Transfection of Pre-miR-31 and Pre-miR-155 (individually or combined) significantly reduced the expression of *IL13RA1* mRNA and protein compared to Control (Figure 5a,b and Figure S2).

We also evaluated the activation of the IL-13 pathway, as reflected by the IL-13-dependent phosphorylation of STAT6, the main signalling mediator of IL-13 [13]. We transfected HT-29 gut epithelial cells with miR mimics as above and stimulated the cells with IL-13. The IL-13-dependent phosphorylation of STAT6 was reduced by miR-31 and miR-155 (Figure 5c and Figure S3) as shown by Western blot analysis. We employed both suppressor of cytokine signalling 1 (*SOCS1)* and epithelial eotaxin-3 (*CCL26)* mRNA evaluation as reporters of IL-13-dependent transcriptional activation. Reverse transcription and real-time PCR analysis showed that expression of *SOCS1* and *CCL26* mRNA was down regulated by miR-31 and miR-155 separately and in combination (Figure 5d). Control levels without IL-13 treatment are shown in Figure S4.

To confirm the effects of miR-31 and miR-155 on *IL13RA1* expression in human colonic tissue, we used an ex vivo explant culture system. Inflamed colonic tissue from patients with UC (Table 4) was directly transfected with Pre-miR-31 or Pre-miR-155 individually (100 nM) or in combination (50 nM each) and compared to 100nM scrambled miR control-transfected biopsies (Control). We harvested the samples 24 h post-transfection and determined mRNA expression of *IL13RA1*. Figure 5e shows that miR-31, miR-155 and their combination were able to significantly decrease the expression of *IL13RA1* mRNA compared to biopsies transfected with scrambled miR control. Our data suggests that increased miR-31 and miR-155 may exert a suppressive role against IL-13-dependent effects in the pathophysiology of UC by reducing IL13Rα1 levels.

## 4. Discussion

Our work demonstrates a role for miR-31 and miR-155 in the regulation of IL-13 signalling. We show for the first time that the main receptor for IL-13, IL13Rα1, is significantly decreased in inflamed colonic tissue from patients with UC (Figure 1). As another novel finding we show that miR-31 is upregulated in active UC compared to inactive UC or healthy donors and directly targets the 3′UTR of *IL13RA1* mRNA. MicroRNA-31 and miR-155 individually and in combination are able to significantly decrease *IL13RA1* expression (Figure 5a,b) and IL-13-dependent responses as reflected by decreased IL-13-dependent phosphorylation of STAT6 (Figure 5c) as well as mRNA expression of *SOCS1* and *CCL26* in gut epithelium (Figure 5d). We acknowledge that the effect on *SOCS1* mRNA downregulation by miR-155 may be partially due to direct targeting of its 3′UTR ([33] and Figure S4). However, this reduction was only 20% in the absence of IL-13 and more than 50% after IL-13 activation, suggesting that the blocking of IL-13 signalling was the most important component of the reduction in *SOCS1* levels by miR-155. We observed a reduction in the IL-13 effect on *SOCS1* and *CCL26* mRNA expression when miR mimics were used (Figure 5d), but we did not observe an additive effect of miR-31 and miR-155 in combination. This suggests that these two miRs are important in UC but work individually, rather than synergistically in regards to *IL13RA1* regulation. Downregulation of IL13Rα1 may thus reduce signalling by IL-13 in gut epithelium and thus block the contribution of IL-13 to UC pathogenesis.

Our results may be of relevance in other disease settings where IL-13-driven processes in the epithelium play a key role. For example, reduction of IL13Rα1 expression by miR-143 has been shown to reduce IL-13-induced mucus production in nasal epithelial cells from patients with allergic rhinitis [34]. It was also recently demonstrated that IL13Rα1 expression is diminished in lung sections from patients with interstitial pulmonary fibrosis [35]. This work showed that *IL13RA1* may have a protective role in lung injury and repair, as *Il13ra1* knock out mice display an increase in bleomycin-induced lung fibrosis [35]. 

Exogenous miR mimics of miR-31 and miR-155 can actively downregulate *IL13RA1* expression in gut epithelial cells in vitro but also importantly in ex vivo explant cultures from inflamed colonic mucosa from patients with UC (Figure 5e). Our data therefore support a key role for miR-31 and miR-155 in UC via directly targeting of *IL13RA1* mRNA. As it happens in the lung [35], IL-13 may play a protective role against fibrosis in the gut and thus miR-31 and miR-155 may have a pathological role by targeting *IL13RA1.*

An alternative explanation is that the downregulation of *IL13RA1* by these miRs in UC may be a feedback mechanism to limit tissue damage initially caused by IL-13 [9,11] and the increase of IL-13 activated genes such as *SOCS1* [36] and *CCL26* [37] in the colon of patients with UC. Although two phase II trials with monoclonal antibodies against IL-13 [38,39] in moderate to severe UC were not significantly superior to placebo, a subset of patients seemed to benefit from blockade of IL-13 [38,40]. We assessed the levels of IL-13 (Figure S5) and observed a decreased presence of IL-13 cytokine in colonic biopsies from patients with active UC (Figure S5). Although these findings need to be corroborated on an expanded cohort, the levels of IL-13 in patients with UC remains controversial. Our interpretation is that these data may explain the failure of anti-IL-13 trials, and that IL-13 levels are not directly responsible for *IL13RA1* regulation. It is possible that IL-13 expression peaks only at certain stages (e.g., during a disease flare), with mechanisms in place to reduce its levels but its effects long-lasting, which we are able to observe as a feedback mechanism. This is particularly relevant since a recent study demonstrates that mice develop UC-like pathology in the absence of IL4R in a IL-13-dependent manner, suggesting an important role for IL-13-dependent inflammation in the development of UC [41]. Moreover, inhibiting downstream pathways of the IL13Rα1 shows promising results, highlighting its importance. IL-13 binds to the IL13Rα1 leading to phosphorylation of JAK1, JAK2 and tyrosine kinase 2 (Tyk2) in colon epithelial cells [42]. A recent phase III clinical trial in UC shows that inhibition of JAK1 and JAK3 with Tofacitinib leads to significant clinical remission rates and mucosal healing in patients with active UC [43]. These observations indicate that the IL-13 signalling plays a role in the homeostasis of the gut mucosa that is yet to be fully established in humans. Thus, while we have demonstrated that miR-31 and miR-155 can regulate IL-13 signalling, the precise role of these miRs in the pathophysiology of UC will require the elucidation of the impact of IL-13 in the human gut. Future work will establish whether miR-31 and miR-155 play also a role in other IL-13 related diseases such as asthma and atopic dermatitis as well as targeting other genes involved in UC.

In summary, we have described a novel mechanism by which increased levels of miRs in UC, namely miR-31 and miR-155, regulate the IL-13 pathway. Thus, these miRs may be used in the future as biomarkers or therapeutic targets to restore or block the IL-13 signalling in diseases such as asthma, atopic dermatitis, eosinophilic esophagitis and UC.

## Figures and Tables

**Table 1:** Demographic data of patients for paired mRNA and microRNA (miR) analysis. *n:* number of subjects; n/a: not applicable; St. dev.: standard deviation; 5-aminosalicylic acid: 5-ASA.

	*Active ulcerative Colitis (UC) (n = 11)*	*Normal Colon (n = 11)*
*Average age (years)*	47.3 (range 22–85)	56.1 (range 46–78)
*Sex*	Male 6; Female 5	Male 6; Female 5
*Duration of disease; Mean (range)*	10.0 years (1–34)	n/a
*Extent of disease*	Left sided colitis 6; Distal colitis 5	n/a
*Mayo score; Mean (St. dev.)*	Clinical 7.55 (2.12); Endoscopic 2.73 (0.47)	0
*5-ASA*	6	n/a
*Thiopurines*	3 (2 also on 5-ASA)	n/a
*Infliximab/Adilumimab*	1 (also on 5-ASA)	n/a
*No medication*	3	n/a

**Table 2:** Demographic data of patients analysed by Western blot. *n:* number of subjects; St. dev.: standard deviation; 5-aminosalicylic acid: 5-ASA.

	*UC Inactive (n = 6)*	*UC Active (n = 6)*
*Average age (years)*	42.3 (range 23–69)	40.1 (range 20–72)
*Sex*	Male: 3/Female: 3	Male: 3/Female: 3
*Duration of disease; Mean (range)*	12.6 years (1–36)	14.4 years (1–31)
*Extent of disease*	Left sided colitis 5; Distal colitis 1	Pan-colitis 3; Left sided colitis 3
*Mayo score; Mean (St. dev.)*	0.5	2.5
*5-ASA*	2	2(6)
*Thiopurines*	2 (2 also on 5-ASA)	2 (2 also on 5-ASA)
*No medication*	3	3

**Table 3:** Demographic data of patients used for epithelial cell isolation. *n:* number of subjects; St. dev.: standard deviation; 5-aminosalicylic acid: 5-ASA.

	*UC Inactive (n = 5)*	*UC Active (n = 5)*
*Average age (years)*	39.3 (range 23–71)	42.1 (range 21–67)
*Sex*	Male: 3/Female: 2	Male: 2/Female: 3
*Duration of disease; Mean (range)*	10.6 years (1–34)	12.2 years (1–41)
*Extent of disease*	Left sided colitis 4; Distal colitis 1	Pan-colitis 3; Left sided colitis 2
*Mayo score; Mean (St. dev.)*	0.4 (0.5)	2.6 (0.5)
*5-ASA*	2	2
*Thiopurines*	1 (1 also on 5-ASA)	1 (1 also on 5-ASA)
*No medication*	2	2

**Table 4:** Demographic data of patients used for explant culture analysis. *n:* number of subjects; St. dev.: standard deviation; 5-aminosalicylic acid: 5-ASA.

	*Active UC (n = 7)*
*Average age (years)*	47.3 (range 20.2)
*Sex*	Male: 4/Female: 3
*Duration of disease; Mean (range)*	Years 11.6 (range 10.6)
*Extent of disease*	Left sided colitis 3/Pancolitis 4
*Mayo score; Mean (St. dev.)*	Clinical 7.88 (2.12); Endoscopic 2.64 (0.47)
*5-ASA*	3 (7)
*Azathioprine/6-Mercapropurin*	2 (7) (2 also on 5-ASA)
*Infliximab/Adilumimab*	0 (7)
*No medication*	2 (7)

## Supplementary Materials

The following are available online at http://www.mdpi.com/2073-4425/9/2/85/s1, Figure S1: Western blotting of IL13Rα1 colonic biopsies; Figure S2: Representative Western blotting detection of IL13Rα1 in HT-29 cells transfected with pre-miR-31, pre-miR-155 or a combination of both; Figure S3: Representative Western blotting detection of phospho-STAT6 in HT-29 cells transfected with pre-miR-31, pre-miR-155 or a combination of both (pre-miR-31/155); Figure S4: Effects of miR-31, miR-155 and their combination on the expression of *SOCS1* mRNA in an IL-13-dependent and independent manner in HT-29 cells; Figure S5: IL-4 and IL-13 cytokine expression from colonic biopsies from patients with inactive or active UC compared to normal non-UC controls. Table S1: Predicted positions (TargetScan) of microRNA binding sites in the 3′UTR of *IL13RA1*. Table S2: Demographic data of patients ELISA for IL-13 and IL-4 (Figure S5).
