# Supplementary material for: MicroRNA-31 and MicroRNA-155 Are Overexpressed in Ulcerative Colitis and Regulate IL-13 Signaling by Targeting Interleukin 13 Receptor α-1
Source: Genes (Basel). 2018 Feb 13;9(2):85. doi: 10.3390/genes9020085 (PMC5852581; doi:10.3390/genes9020085)
Supplement: Supplementary file 1 [file genes-09-00085-s001.docx]

SUPPLEMENTARY DATA FOR

# MicroRNA-31 and microRNA-155 are overexpressed in Ulcerative Colitis and Regulate IL-13 Signaling by Targeting Interleukin 13 Receptor α-1

**Markus Gwiggner, Rocio T. Martinez-Nunez, Simon R. Whiteoak Victor P. Bondanese, Andy Claridge, Jane E. Collins, JR Fraser Cummings, Tilman Sanchez-Elsner**

| MicroRNA | Position |
| --- | --- |
| miR-27a-3p | 25-31 |
|  | 2217-2223 |
| miR-31-5p | 1158-1165 |
| miR-152-3p | 2560-2566 |
| miR-155-5p | 1049-1071 |
|  | 1399-1424 |
| miR-183-5p | 1556-1562 |
| miR-324-3p | 975-981 |
| miR-374a-5p | 215-221 |

**Table S1:** Predicted positions (TargetScan) of microRNA binding sites in the 3’UTR of *IL13RA1*.


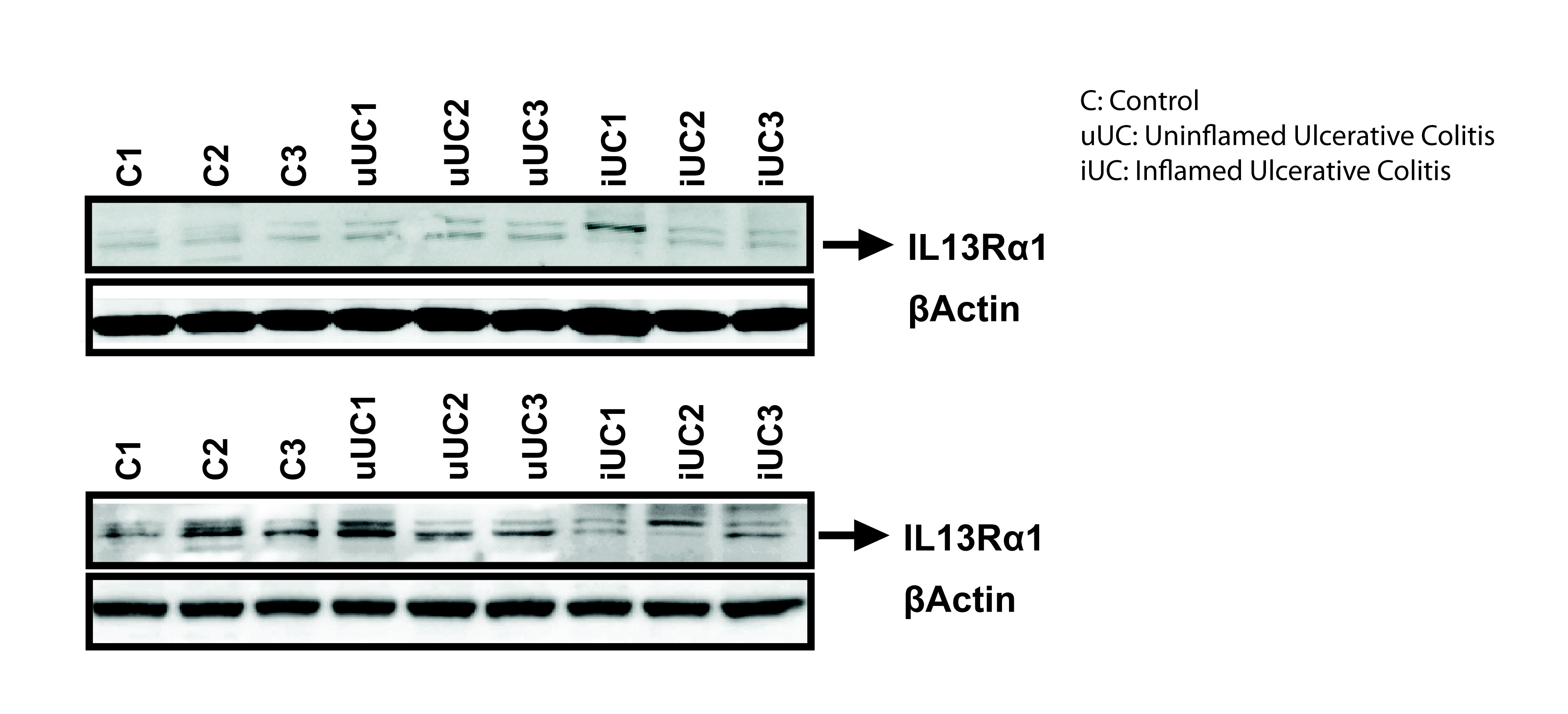


**Figure S1:** Western blotting of IL13Rα1 colonic biopsies. Blots depicting the expression of IL13Rα1 protein in colonic biopsies. C: healthy controls; uUC: uninflamed colonic biopsies; iUC: inflamed colonic biopsies (n=6 each); βActin: beta-actin, loading control. Full original blots are shown below. IL13Rα1 protein band selected as shown in “Upper Blots” according to previous publication.


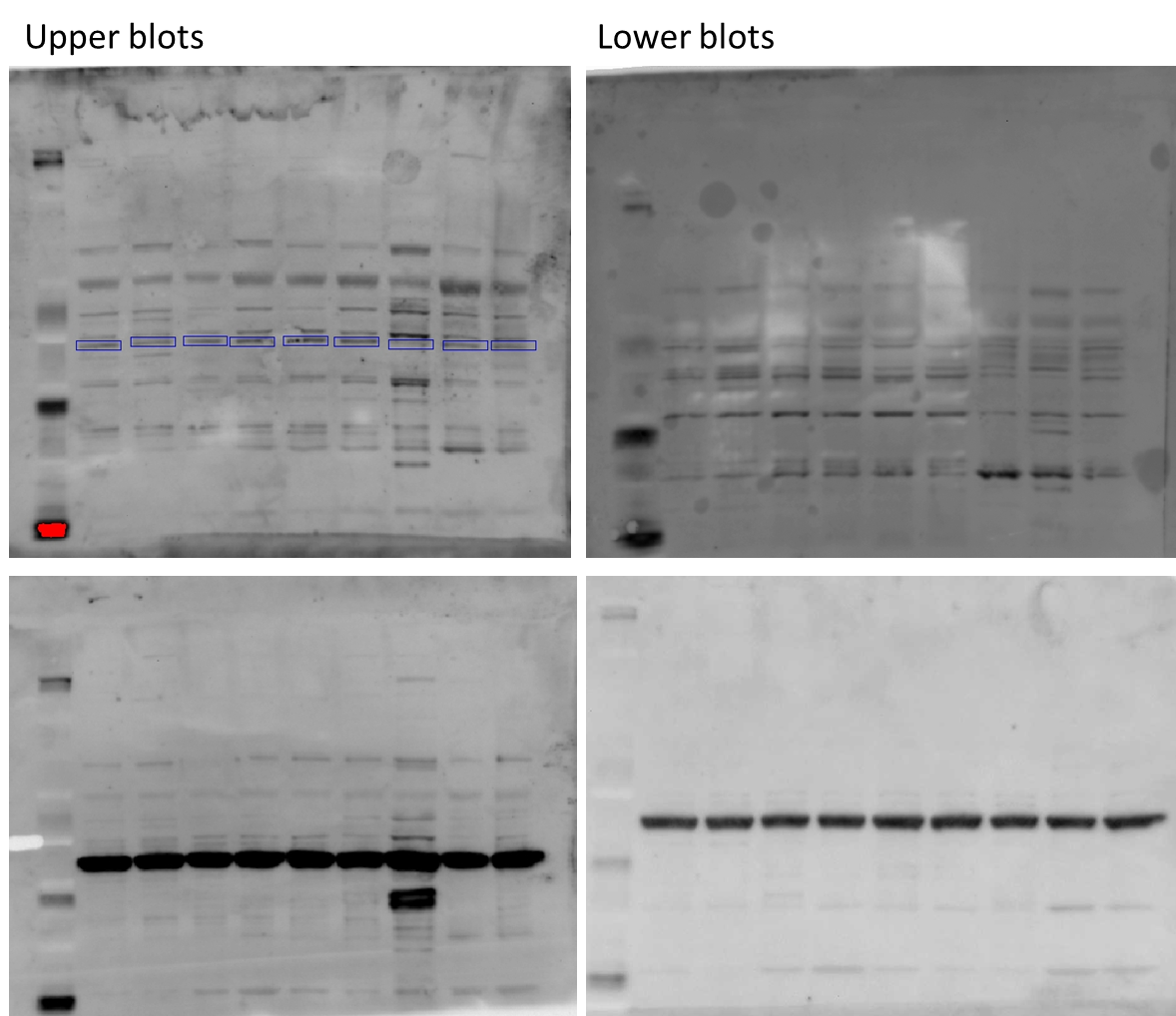

**Figure S2:** Representative Western blotting detection of IL13Rα1 in HT-29 cells transfected with pre-miR-31, pre-miR-155 or a combination of both (premiR-31/155). Full original blots are shown below.


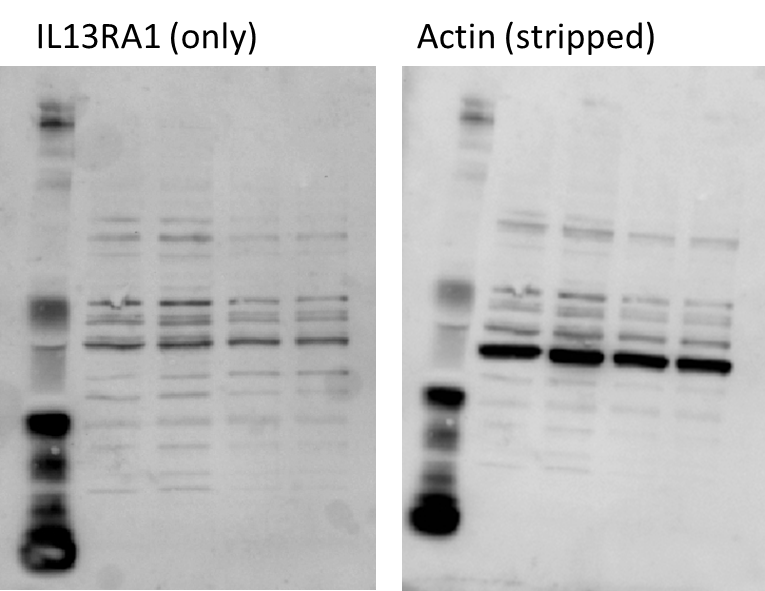


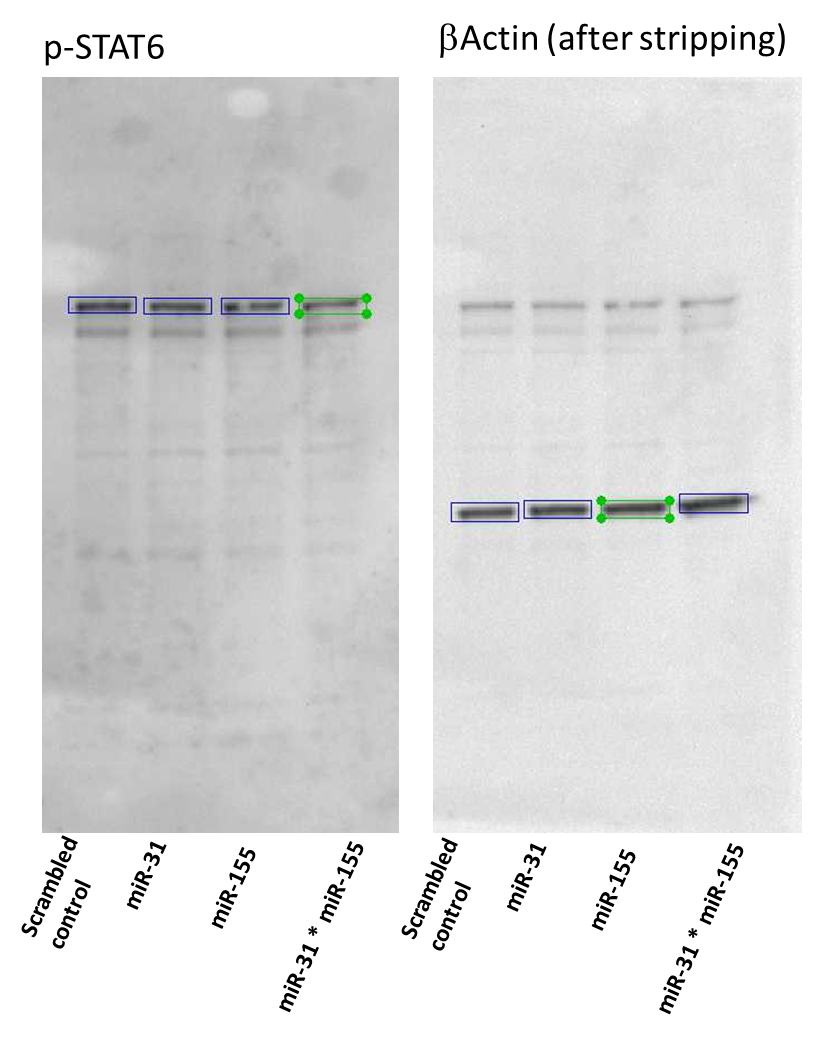
 **Figure S3:** Representative Western blotting detection of phospho-STAT6 in HT-29 cells transfected with pre-miR-31, pre-miR-155 or a combination of both (pre-miR-31/155). Boxes show the bands used for densitometry quantification.


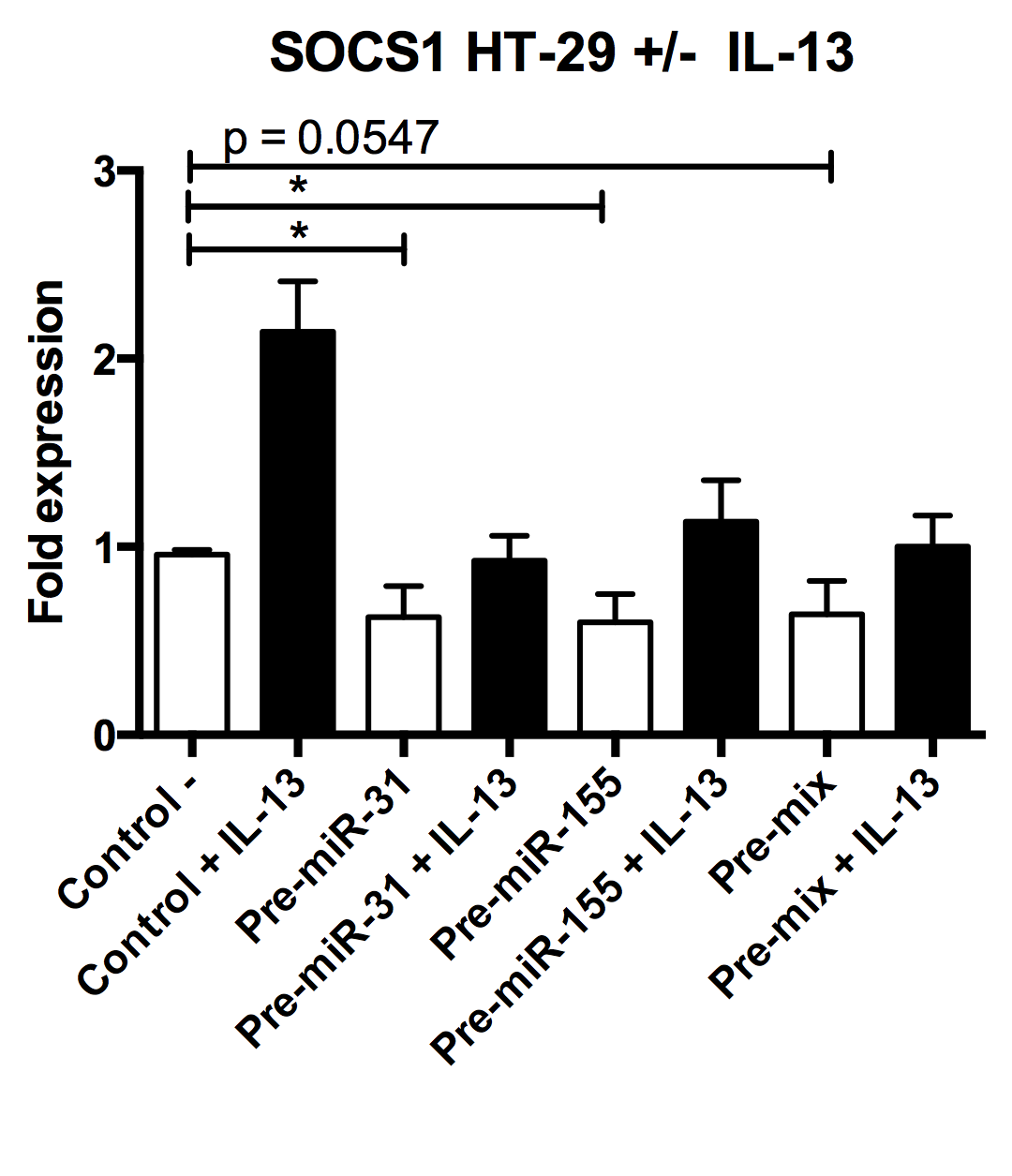


**Figure S4:** Effects of miR-31, miR-155 and their combination on the expression of *SOCS1* mRNA in an IL-13-dependent and independent manner in HT-29 cells. Graph bar depicting *SOCS1* mRNA expression in HT-29 cells transfected with pre-miR-31, pre-miR-155 or a combination of both (premiR-31/155) (n=8).

**Figure S5:** IL-4 and IL-13 cytokine expression from colonic biopsies from patients with inactive or active UC compared to normal non-UC controls. *: p<0.05 and *** p<0.001

|  | **UC inactive (N=8)** | **UC active (N=8)** |
| --- | --- | --- |
| **Average Age** | 42.3 (range 23-69) | 40.1 (range 20-72) |
| **Sex** | Male: 3/Female: 3 | Male: 3/Female: 3 |
| **Duration of disease** | Years 12.6 (range 1-36) | Years 14.4 (range 1-31) |
| **Extent of disease** | Left sided colitis 5  Distal colits 1 | Pan-colitis 3  Left sided colits 3 |
| **Endoscopic Mayo score** | 0.5 (STDEV0.5) | 2.5 (STDEV0.5) |
| **5-ASA** | 2 | 2(6) |
| **Thiopurines** | 2 (2 also on 5-ASA) | 2 (2 also on 5-ASA) |
| **No medication** | 3 | 3 |

**Table S2:** Demographic data of patients ELISA for IL-13 and IL-4 (FigS5).
